# Whole‐exome sequencing for genetic diagnosis of idiopathic liver injury in children

**DOI:** 10.1111/jcmm.18485

**Published:** 2024-06-12

**Authors:** Aysima Atılgan Lülecioğlu, Yılmaz Yücehan Yazıcı, Alperen Baran, Khaled Warasnhe, Şengül Beyaz, Caner Aytekin, Figen Özçay, Yusuf Aydemir, Zeren Barış, Serkan Belkaya

**Affiliations:** ^1^ Department of Molecular Biology and Genetics, Faculty of Science İhsan Doğramacı Bilkent University Ankara Turkey; ^2^ Department of Pediatrics Başkent University Faculty of Medicine Ankara Turkey; ^3^ Department of Immunology and Allergy Diseases Ankara Bilkent City Hospital Ankara Turkey; ^4^ Department of Pediatric Immunology Dr. Sami Ulus Maternity and Children's Health and Diseases Training and Research Hospital Ankara Turkey; ^5^ Department of Pediatric Gastroenterology and Hepatology Başkent University Faculty of Medicine Ankara Turkey; ^6^ Department of Pediatric Gastroenterology, Faculty of Medicine Eskişehir Osmangazi University Eskişehir Turkey

**Keywords:** acute liver failure, children, idiopathic hepatic injury, recurrent elevated transaminases, whole‐exome sequencing

## Abstract

Genome‐wide approaches, such as whole‐exome sequencing (WES), are widely used to decipher the genetic mechanisms underlying inter‐individual variability in disease susceptibility. We aimed to dissect inborn monogenic determinants of idiopathic liver injury in otherwise healthy children. We thus performed WES for 20 patients presented with paediatric‐onset recurrent elevated transaminases (rELT) or acute liver failure (ALF) of unknown aetiology. A stringent variant screening was undertaken on a manually‐curated panel of 380 genes predisposing to inherited human diseases with hepatobiliary involvement in the OMIM database. We identified rare nonsynonymous variants in nine genes in six patients (five rELT and one ALF). We next performed a case‐level evaluation to assess the causal concordance between the gene mutated and clinical symptoms of the affected patient. A genetic diagnosis was confirmed in four rELT patients (40%), among whom two carried novel mutations in *ACOX2* or *PYGL*, and two had previously‐reported morbid variants in *ABCB4* or *PHKA2*. We also detected rare variants with uncertain clinical significance in *CDAN1*, *JAG1*, *PCK2*, *SLC27A5* or *VPS33B* in rELT or ALF patients. In conclusion, implementation of WES improves diagnostic yield and enables precision management in paediatric cases of liver injury with unknown aetiology, in particular recurrent hypertransaminasemia.

## 1 INTRODUCTION

Childhood liver disorders can be upon a variety of internal and external culprits including infections, drugs and toxins, metabolic disorders, autoimmunity and malignancy, with severity of injury ranging from mild, transient elevations in liver enzymes to severe hepatic failure.^1^, ^2^, ^3^, ^4^, ^5^, ^6^, ^7^ Affected children with poorly functioning liver may need prolonged treatment and/or transplantation, or even die. Yet, early diagnosis of acute liver disease is still challenging for paediatric hepatologists, often due to the absence of specific symptoms or the presence of nonspecific clinical manifestations; thus, despite extensive diagnostic workup, nearly 50% of paediatric acute liver failure (ALF) cases remain undiagnosed.^1^, ^2^, ^3^, ^4^, ^5^, ^6^, ^7^, ^8^ Next‐generation sequencing technologies, such as whole‐exome and ‐genome sequencing, are now widely used in clinics as powerful genome‐wide scanning tools in the search for genetic variants responsible for phenotypes of interest.^9^, ^10^, ^11^ Recent studies have highlighted the utility of whole‐exome sequencing (WES) in the identification of genetic causes underlying liver diseases of unknown aetiology in both paediatric and adult patients.^12^, ^13^, ^14^, ^15^, ^16^, ^17^, ^18^, ^19^, ^20^, ^21^, ^22^, ^23^ However, genetic studies focusing on children and, in particular, patients with different ethnic backgrounds are still limited. The identification of inborn monogenic causes of indeterminate liver diseases holds significant clinical importance, as it may facilitate familial testing for earlier diagnosis, prognosis prediction and more precise management in high‐risk family members. Genetic diagnosis streamlines treatment modalities, enhances medical management and clinical decision‐making for transplantation. Therefore, in this study, we aimed to identify candidate disease‐causing single‐gene variants by WES in patients with childhood‐onset recurrent idiopathic hypertransaminasemia or with indeterminate paediatric ALF.

## 2 MATERIALS AND METHODS

### 2.1 Ethics

This study was conducted in accordance with the institutional, local and national ethical guidelines, and approved by the İhsan Doğramacı Bilkent University Ethics Committee (#2019_11_21_07 and #2020_06_17_01). Clinical history and peripheral blood samples were obtained by the referring physician, with a written informed consent from each participant enrolled in this study and parents if the participant was a minor.

### 2.2 Patient recruitment

Patient recruitment into this study was performed together with a network of referring physicians in Turkey. Study population included both prospective and retrospective cases with paediatric‐onset (≤18 years of age) idiopathic liver injury. The evaluation and final diagnosis of the cases were determined entirely at the referring physician's discretion. If the physician could not establish a specific diagnosis for the cause of liver dysfunction, due to lack of supporting evidence, the final diagnosis was considered as idiopathic. Overall, idiopathic cases were enrolled within two diagnostic groups: (i) recurrent elevated liver transaminases (rELT) and (ii) ALF. For rELT, we recruited patients who have had paediatric‐onset, at least three repeated episodes of elevated liver transaminases (alanine aminotransferase [ALT] and aspartate aminotransferase AST), equal to or more than twice the upper limit of normal range, with normalization of liver biochemical parameters between crises. For ALF, we recruited patients based on widely accepted criteria including (i) biochemical evidence of acute liver injury with no evidence of chronic liver disease and (ii) hepatic‐based coagulopathy not corrected by vitamin K administration (international normalized ratio (INR) ≥1.5 with hepatic encephalopathy or INR ≥2.0 without hepatic encephalopathy).^1^, ^2^, ^3^

### 2.3 WES and variant analysis

WES was performed on the genomic DNA (gDNA) isolated from the peripheral blood samples of the participants using commercially available kits. Library preparation, collection of raw sequencing data, alignment with the reference human genome, variant calling and annotations were performed by a WES service provider, Macrogen, Europe or Genoks, Turkey. The allele frequency (AF) values were obtained from the public genome databases: gnomAD,^24^ Bravo,^25^ and UK Biobank‐Allele Frequency Browser (AFB).^26^ Low quality variants were removed. Only predicted loss‐of‐function (pLOF) (frameshift indel, stop‐gain and essential splicing [± 2 bp from the exon‐intron boundary]), start‐loss, stop‐loss, in‐frame indel and missense variants were retained for further analysis. The damaging impact of variants was in silico predicted using various algorithms including Polymorphism Phenotyping v2 (PolyPhen‐2) and Sorting intolerant from tolerant (SIFT) for missense variants,^27^, ^28^ and MutationTaster2021 and Combined annotation‐dependent depletion (CADD) v1.6 together with Mutation Significance Cut‐off (MSC) for both single nucleotide variants and indels.^29^, ^30^, ^31^ Regarding CADD, the variant was considered damaging if the CADD score was higher than the MSC value (95% confidence interval) of the mutated gene. Significance of the variants related to the human health were obtained from the ClinVar database (https://www.ncbi.nlm.nih.gov/clinvar/). Clinical interpretation and classification of sequence variants were performed manually and using an automated tool such as InterVar,^32^ based on the American College of Medical Genetics and Genomics (ACMG) and the Association for Molecular Pathology (AMP) guidelines.^33^ Details of WES analysis and variant filtering are provided in the Supplemental Materials and Methods.

### 2.4 Sanger sequencing of gDNA

The regions encompassing the target alleles on gDNA were amplified by PCR. Sanger sequencing of the PCR amplicons were performed by a service provider (Macrogen). SnapGene Viewer software (GSL Biotech LLC, USA) was used for sequence analysis. Primers are listed in Table S1.

## 3 RESULTS

### 3.1 Characteristics of the study population

We recruited a total of 20 patients with idiopathic liver injury: 10 patients with recurrent elevated liver transaminases (rELT) and 10 patients with ALF. All cases were sporadic with no familial history. Female and male patients were nearly equally distributed in both groups. Eight patients, six rELT and two ALF, were born to consanguineous parents. All the rELT patients were alive as of the writing of this article. Among the ALF patients, seven underwent transplantation and two recovered upon medical treatment. Two ALF patients died. WES was performed for all 20 patients, with 13 singletons (five patients with rELT and eight patients with ALF) and seven patient‐parent trios (five trio designs with rELT and two trio designs with ALF).

### 3.2 Search for variants in genes predisposing to inherited diseases with liver involvement

**FIGURE 1:**
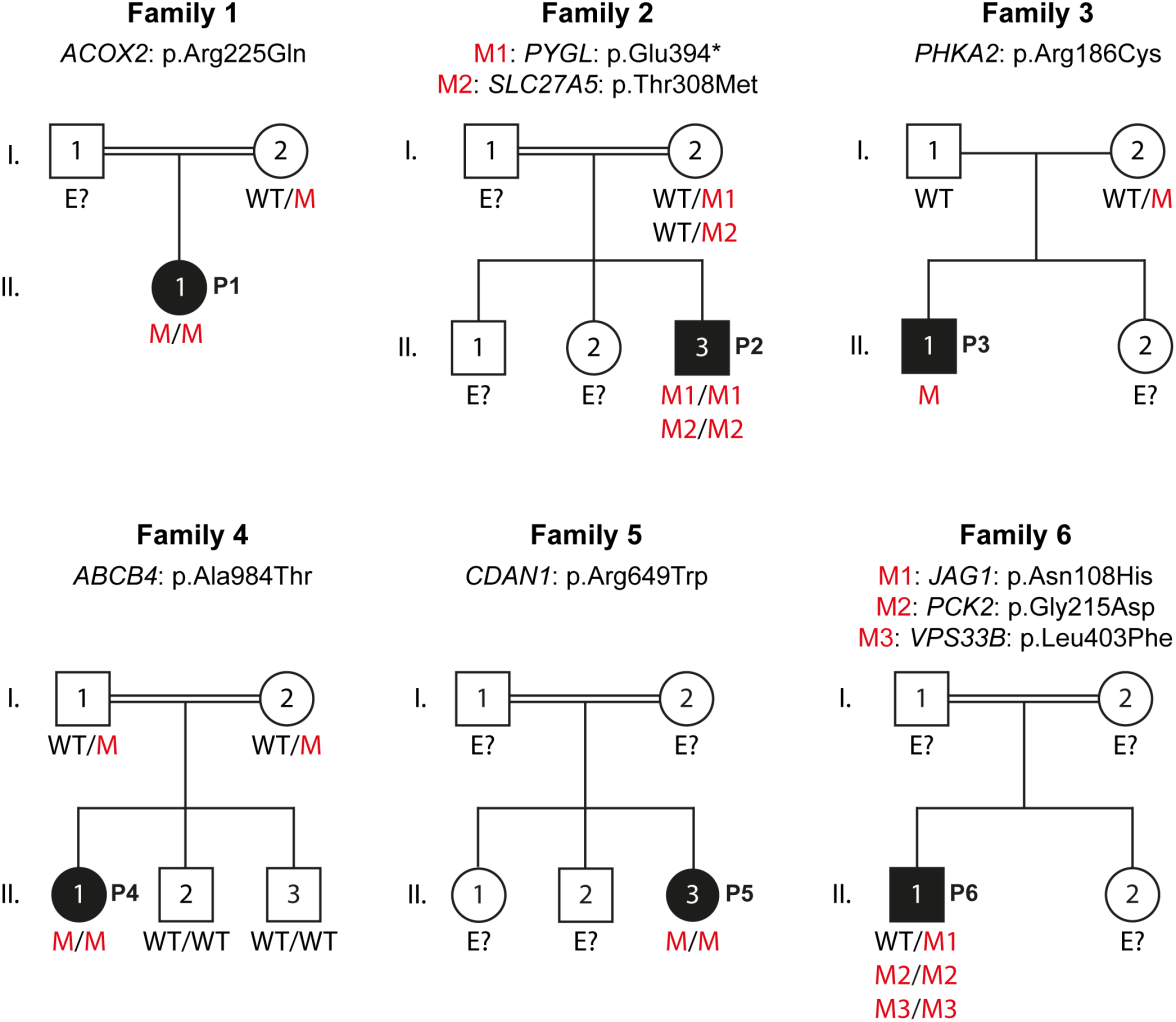
Familial pedigrees. Pedigrees of the six families affected by idiopathic liver injury with nonsynonymous variants in the liver panel genes are shown. Patients (P1–P6) are shown in black, whereas healthy individuals are shown in white. Familial segregation of the variants with the disease was confirmed by Sanger sequencing. Mutation status is indicated, where possible. WT, wild type, M, mutation.

**FIGURE 2:**
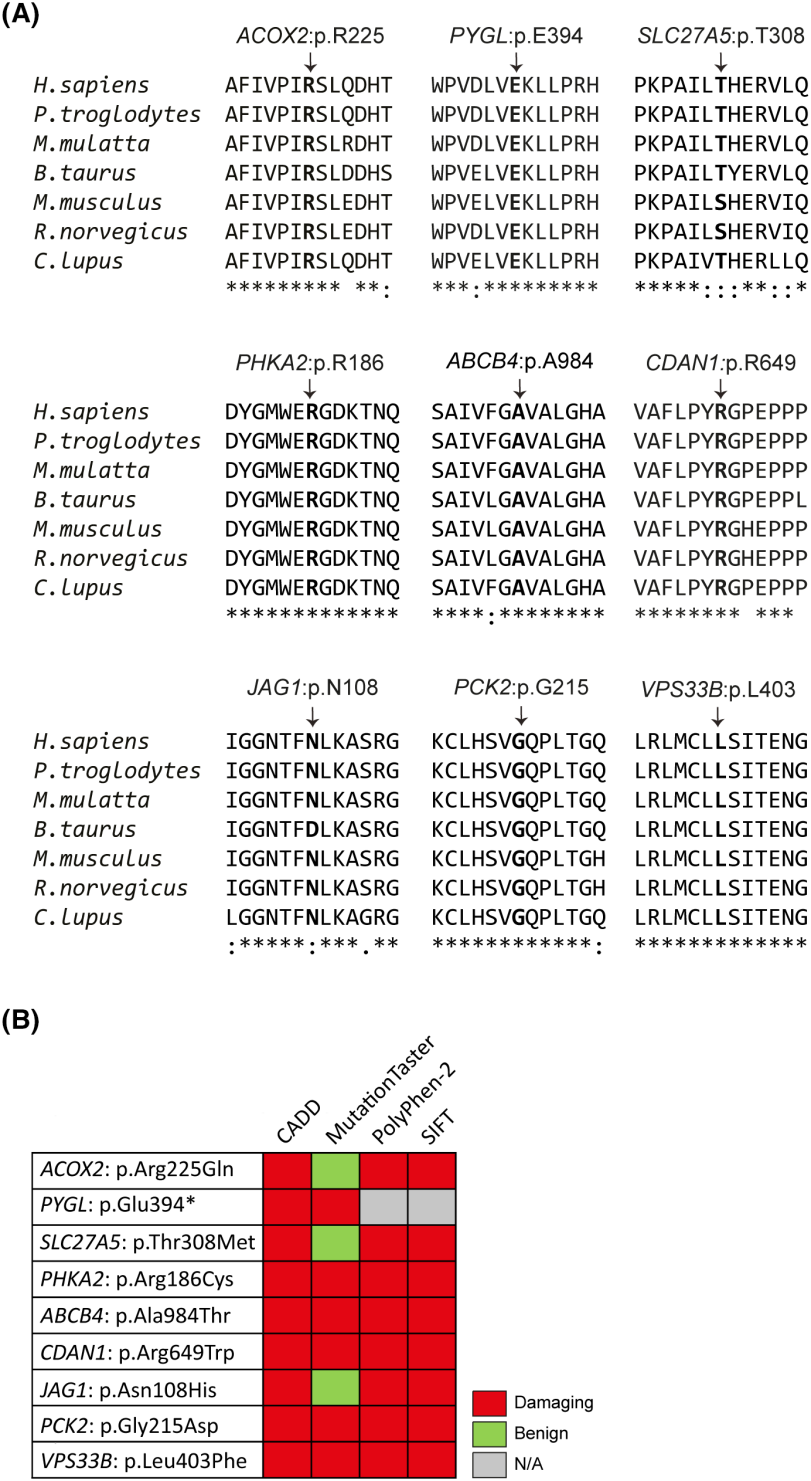
Variant effect predictions. (A) Schemas show conservation of mutated amino acid residues across various species. Asterisk (*), colon (:), and period (.) indicate fully conserved, strongly similar, and weakly similar sites, respectively. Source: Clustal Omega (B) The predicted impact of variants using four algorithms, CADD, MutationTaster, PolyPhen‐2 and SIFT, is shown. Red colour indicates damaging, whereas green colour is benign. N/A, Not applicable.

We performed a biased WES analysis to determine whether there were any candidate pathogenic variants in genes previously reported to be associated with inherited diseases with liver involvement. We therefore manually curated a liver gene panel by searching for the genes annotated with hepatic and/or biliary phenotype or laboratory finding of elevated liver enzymes/transaminases in the OMIM database (https://www.omim.org/). A total 380 genes were included in the liver panel, which is listed in Table S2. We analysed the WES data of all patients to search for (i) biallelic (homozygous and compound heterozygous) variants with AF <1% in liver panel genes linked with autosomal recessive (AR) or X‐linked recessive (XLR) modes of inheritance and (ii) monoallelic (heterozygous and hemizygous) variants with AF <0.01% in liver panel genes linked with autosomal dominant (AD), X‐linked dominant, or XLR modes of inheritance, respectively. Variants listed as benign in the ClinVar were excluded. Missense variants predicted to be benign by all in silico prediction algorithms, CADD, MutationTaster, PolyPhen‐2 and SIFT, were also filtered out. Finally, we performed a case‐level review to assess the pathogenicity of the gene mutated with regard to its compatibility with the clinical symptoms of the affected patient. Overall, we identified six (five with rELT and one with ALF) of 20 patients carrying rare nonsynonymous variants in nine genes from the liver panel (Table 1; Figure 1). All variants were located on highly‐conserved amino acid residues across various species (Figure 2A) and predicted to be damaging by at least three of the algorithms tested, including CADD, MutationTaster, PolyPhen‐2 and SIFT, when applicable (Figure 2B). Detailed clinical and laboratory findings of these six patients are provided in Table 2.

**TABLE 1:** Patients with rare nonsynonymous variants in liver panel genes.

Patient	Disease	Gene	Variation					
			Type	Change	Status	AF	ACMG‐AMP	
							Classification	Criteria
P1	rELT	*ACOX2*	Missense	NM_003500.4:c.674G > A:p.Arg225Gln	Hom	5.14E‐05	VUS	PM1, PP3, PM5
P2	rELT	*PYGL*	Nonsense	NM_002863.5:c.1180G > T:p.Glu394*	Hom	‐	P	PVS1, PM2, PP3
		*SLC27A5*	Missense	NM_012254.3:c.923C > T:p.Thr308Met	Hom	1.86E‐05	VUS	PM1, PM2, PP3
P3	rELT	*PHKA2*	Missense	NM_000292.3:c.556C > T:p.Arg186Cys	Hem	9.26E‐07	P	PS1, PM1, PM2, PP3, PP5
P4	rELT	*ABCB4*	Missense	NM_000443.4:c.2950G > A:p.Ala984Thr	Hom	1.37E‐06	LP	PS1, PM2, PP3, BP1
P5	ALF	*CDAN1*	Missense	NM_138477.2:c.1945C > T:p.Arg649Trp	Hom	3.28E‐05	LP	PS1, PM2, PP3
P6	rELT	*JAG1*	Missense	NM_000214.3:c.322A > C:p.Asn108His	Het	2.05E‐06	VUS	PM2, PP3, BP1
		*PCK2*	Missense	NM_004563.4:c.644G > A:p.Gly215Asp	Hom	2.01E‐04	VUS	PM1, PM2, PP3, BP1
		*VPS33B*	Missense	NM_018668.5:c.1209G > T:p.Leu403Phe	Hom	1.37E‐06	VUS	PM1, PM2, PP3, BP1

Abbreviations: AF: overall allele frequency in gnomAD v4.1.0 (as of May 08, 2024); Hom, Homozygous, Hem, Hemizygous, Het: Heterozygous, VUS, variant of uncertain significance, P: Pathogenic, LP: Likely pathogenic.

**TABLE 2:** Clinical and laboratory findings of 6 patients with rare nonsynonymous variants in liver panel genes.

		P1	P2	P3	P4	P5	P6
Sex		Female	Male	Male	Female	Female	Male
Consanguinity		Yes	Yes	No	Yes	Yes	Yes
Delayed growth or development		No	No	No	No	No	No
Age of disease onset		7 months	8 months	12 months	13 years	13 months	7 years
Clinical symptoms at admission		Asymptomatic	Asymptomatic	Asymptomatic	Abdominal pain	Vomiting, fever, diarrhoea	Asymptomatic
ELT episodes	Encephalopathy	No	No	No	No	No	No
	ALT (U/L) (min‐max)	86–885	80–570	93–1121	105–174	6530	92–169
	AST (U/L) (min‐max)	95–1226	90–500	82–2067	108–181	8890	72–140
	ALP (U/L)	Normal	Normal	Normal	Normal	Normal	Normal
	GGT (U/L) (min‐max)	Normal	Variable (Elevated range: 76–109)	Variable (Elevated range: 216–280)	Elevated (70–154)	Normal	Mostly normal (Elevated: 38)
	PT (sec)/INR	Normal / Normal	Normal / Normal	Mostly normal (Elevated: 15 / 1.3)	Variable (Elevated range: 15.1–16.5 / 1.26–1.43)	34 / 3.22	Normal / Normal
	Direct/Indirect bilirubin (mg/dL)	Normal	Normal	Normal	Normal	Normal	Normal
Organomegaly		No	Hepatomegaly	Hepatomegaly	Hepatosplenomegaly	No	No
Autoantibodies*		Negative	Negative	Negative	ANA (+), others: negative	Negative	Negative
Cytopenia		No	No	No	No	No	No
Infectious diseases		No	No	No	No	No	No
Metabolic workup		Normal	Hyperlipidemia	Normal	Normal	Normal	Normal
Extrahepatic abnormalities		No	No	No	No	No	No
Abdominal ultrasound exam		Liver size, surface, and echogenicity were normal.	Liver was 98 mm in the midclavicular line and showed mildly increased parenchymal echogenicity (hepatosteatosis). The right kidney was normal in size and echogenicity. Left renal pelvis anteroposterior diameter was 28 mm, with Grade‐4 hydronephrosis (mild parenchymal loss).	Liver parenchymal echogenicity and surface were normal. The size of the liver was 15 cm.	Liver was 15 cm and its left lobe was hypertrophic. Increased liver parenchymal echogenicity (grade 1–2). Lymphadenopathy (20 × 10 mm) in the liver hilum. Spleen was 20 cm. Two calculi, each measuring 4 and 9 mm, were seen in the gallbladder lumen.	Liver size, surface, and echogenicity were normal	Liver size, surface, and echogenicity were normal
Liver biopsy		NA	NA	NA	NA	NA	Ground glass appearance of hepatocytes with eosinophilic inclusions were seen.
Treatment		UDCA	No	No	UDCA	Medical treatment	UDCA
Outcome		Alive	Alive	Alive	Alive	Alive	Alive

*Note*: Reference ranges: ALT: <40 U/L for P1‐P3, P5, P6; <27 U/L for P4, AST: <40 U/L for P1‐P3; <47 U/L for P4; <30 U/L for P5 and P6, GGT: <40 U/L for P1‐P3; <23 U/L for P4; <21 U/L for P5 and P6, ALP: 0–300 U/L for P1‐P3; 83–382 U/L for P4; 30–120 U/L for P5 and P6, PT: 9.9–11.8 sec for P1‐P3; 10–14.7 sec for P4; 10–14.5 sec for P5 and P6, INR: 0.8–1.2 for P1‐P6, Direct bilirubin: 0–0.2 mg/dL for P1‐P3; 0–0.5 mg/dL for P4‐P6, Indirect bilirubin: 0–0.9 mg/dL for P1‐P3; 0–1.5 mg/dL for P4; 0–0.7 mg/dL for P5 and P6. *Autoantibody screening: antinuclear antibodies (ANA), extractable nuclear antigen panel, anti‐smooth muscle antibodies, antimitochondrial antibodies, antibodies to liver‐kidney microsome type‐1/2, antibodies to soluble liver antigen, antineutrophil cytoplasmic antibodies, Anti‐Saccharomyces cerevisiae antibodies, Anti‐transglutaminase IgA/IgG, Antigliadin IgA/IgG.

Abbreviations: ALT, alanine aminotransferase, AST, aspartate aminotransferase, ALP, alkaline phosphatase, GGT, gamma‐glutamyl transferase, PT, prothrombin time, INR, international normalized ratio, NA, not available, UDCA, ursodeoxycholic acid.

Homozygous p.Arg225Gln in Acyl‐CoA oxidase 2 (*ACOX2*) was identified in P1 with rELT (Table 1). Variant's status in patient's father was not assessed as his gDNA was not available, but the mother was heterozygote as confirmed by Sanger sequencing (Figures 1 and S1). Biallelic mutations in *ACOX2* were linked to congenital bile acid synthesis defect (MIM: 617308). A different homozygous missense mutation at the same location, p.Arg225Trp, was reported in patients with congenital bile acid synthesis defect,^34^, ^35^ whereas p.Arg225Gln was not listed in the ClinVar or previously associated with a disease. Overall, rELT, low‐normal levels of gamma‐glutamyl transferase (GGT) and favourable response to ursodeoxycholic acid treatment in P1 strongly suggested a bile acid synthesis defect (Table 2), probably due to the homozygosity of predicted‐to‐be deleterious p.Arg225Gln in *ACOX2* (Figure 2B), albeit being classified as variant of uncertain significance (VUS) based on the ACMG‐AMP guidelines (Table 1).

We found a homozygous nonsense variation, p.Glu394*, in Glycogen phosphorylase L (*PYGL*) in P2 with rELT (Table 1). Sanger sequencing confirmed that this variant was heterozygous in patient's mother, but unknown in father as his gDNA was not available (Figures 1 and S1). Biallelic mutations in *PYGL* were shown to cause glycogen storage disease VI (MIM: 232700), represented by increased liver glycogen content and hepatomegaly. The p.Glu394*, a novel pLOF variant not listed in any public genome database (Table 1) or ClinVar, was high likely to be morbid in P2, who also had hyperlipidemia and hepatosteatosis (Table 2). In addition, there was a homozygous missense variant, p.Thr308Met, found in Solute Carrier Family 27 Member 5 (*SLC27A5*) in P2 (Table 1). The p.Thr308Met was heterozygous in patient's mother (Figures 1 and S1). *SLC27A5* is mainly expressed in the liver and involved in the regulation of fatty acid and bile acid metabolism.^36^ It was recently shown that Slc27a5 deficiency led to spontaneous liver fibrosis development in mice.^37^ A homozygous missense mutation (p.His338Tyr) in *SLC27A5* has been implicated in Bile acid conjugation defect in a neonate presented with fibrosis and cholestasis in liver biopsy, however there was no experimental evidence provided.^38^ The p.Thr308Met was not listed in the ClinVar or previously reported to contribute to a disease susceptibility. It was predicted to be damaging by CADD, PolyPhen‐2 and SIFT, but it still remains as VUS (Table 1; Figure 2B). Nevertheless, it is possible that homozygosity of p.Thr308Met in *SLC27A5* and p.Glu394* in *PYGL* both contribute to pathogenesis of rELT in P2 (Table 2).

Hemizygous p.Arg186Cys was found in Phosphorylase kinase regulatory subunit alpha 2 (*PHKA2*) in P3 with rELT (Table 1). Sanger sequencing confirmed that patient's mother was heterozygote for this allele, whereas his father was WT, consistent with XLR inheritance (Figures 1 and S1). Inherited mutations in *PHKA2* have been associated with glycogen storage disease (MIM: 306000), which can present with hepatomegaly and elevated liver enzymes. The p.Arg186Cys was listed as likely pathogenic in the ClinVar (VCV000010535.8) and previously implicated in a patient with X‐linked liver glycogenosis type 2.^39^, ^40^ Therefore, this variant was highly considered to be the genetic cause underlying rELT in P3 (Table 2).

We identified a homozygous missense variant, p.Ala984Thr, in ATP binding cassette subfamily B member 4 (*ABCB4*) in P4 with rELT (Table 1). Biallelic mutations in *ABCB4* have been associated with Gallbladder disease 1 (MIM: 600803) and progressive familial intrahepatic cholestasis type 3 (PFIC3) (MIM: 602347). We confirmed by Sanger sequencing that the familial segregation of the p.Ala984Thr allele was consistent with an AR mode of inheritance, as both healthy parents were heterozygous for the mutation, whereas the healthy siblings did not carry the mutation (Figures 1 and S1). The p.Ala984Thr was previously reported at heterozygosity in an adult patient with PFIC3, albeit without any experimental evidence of causality.^41^ Yet, the homozygosity of *ABCB4*:p.Ala984Thr, predicted as likely pathogenic (Table 1; Figure 2B), could largely explain the clinical symptoms of P4, which included elevated GGT, hepatosplenomegaly and cholelithiasis (Table 2).

A homozygous missense variant, p.Arg649Trp, in Codanin 1 (*CDAN1*) was found in P5 with ALF (Table 1). Zygosity of p.Arg649Trp was not assessed in P5's parents as their gDNA samples were not available (Figures 1 and S1). Biallelic mutations in *CDAN1* have been implicated in congenital dyserythropoietic anaemia type Ia (MIM: 224120), in which affected patients may develop severe hepatic injury such as ALF.^42^, ^43^ Indeed, the p.Arg649Trp with p.Arg397Trp at compound heterozygosity were reported in a patient with congenital dyserythropoietic anaemia type Ia.^44^ However, although this variant was interpreted as likely pathogenic based on the ACMG‐AMP classification (Table 1), P5's clinical findings were not compatible with the known phenotypic spectrum of *CDAN1* deficiency (Table 2).

Last, we identified heterozygous p.Asn108His in Jagged canonical Notch ligand 1 (*JAG1*), homozygous p.Gly215Asp in Phosphoenolpyruvate carboxykinase 2, mitochondrial (*PCK2*) and homozygous p.Leu403Phe in Vacuolar protein sorting 33B, late endosome and lysosome associated (*VPS33B*) in P6 with rELT (Table 1). Familial segregation of these three variants with the disease was not assessed as gDNA samples from parents were not available (Figures 1 and S1). Inherited heterozygous variants in *JAG1* are associated with Alagille syndrome 1 (MIM: 118450), which can include liver phenotypes, such as cholestasis and intrahepatic duct deficiency, and laboratory abnormalities including increased conjugated bilirubin, hypercholesterolemia, hypertriglyceridemia, and elevated transaminases. The p.Asn108His in *JAG1* was listed as VUS in the ClinVar (VCV001020741.7). Moreover, biallelic mutations in *PCK2* have been associated with mitochondrial phosphoenolpyruvate carboxykinase deficiency (MIM: 261650), characterized by hypoglycemia and liver failure. Finally, biallelic mutations in *VPS33B* have been associated with arthrogryposis, renal dysfunction, and cholestasis (MIM: 208085), progressive familial intrahepatic cholestasis (MIM: 620010), and keratoderma‐ichthyosis‐deafness syndrome (MIM: 620009), all of which have liver abnormalities reported. However, both p.Gly215Asp in *PCK2* (VUS in ClinVar, VCV002713587.2) and p.Leu403Phe in *VPS33B* were not previously associated with any known diseases. Yet, none of these three genetic variants of uncertain significance matched with the patient's clinical phenotype (Tables 1 and 2).

## 4 DISCUSSION

Liver injury of unknown origin still represents a major burden in paediatric hepatology despite diagnostic advances and extensive aetiological workup. This may be attributed in part to nonspecific findings and/or overlapping symptoms, presented by a known disease with primary or secondary liver involvement. Alternatively, it could signify a novel liver disorder with yet uncharacterized clinical manifestations. Thus, there is an urgent necessity for enhanced diagnostic approaches to enable earlier and more precise interventions in affected children. In particular, WES has been instrumental in the genetic diagnosis of idiopathic liver diseases.^12^, ^13^, ^14^, ^15^, ^16^, ^17^, ^18^, ^19^, ^20^, ^21^, ^22^, ^23^ Herein, we investigated a total of 20 paediatric cases with rELT or ALF of unknown aetiology using WES. While there was no candidate morbid variation found in ALF patients, we established a genetic diagnosis in four out of 10 rELT patients (40%) using a liver gene panel. Mutations identified in *ACOX2* and *PYGL* in two patients with rELT were novel, thereby expanding the genetic spectrum of the clinical symptoms associated with these genes. Two rELT patients had previously‐described morbid variations in *ABCB4* or *PHKA2*. Overall, our findings, although derived from a relatively small cohort, demonstrate the clinical utility of WES in molecular diagnosis of idiopathic liver injury, especially in patients who present with nonspecific findings such as hypertransaminasemia.

The aetiology of paediatric ALF is multifactorial. Despite diagnostic advances and extensive etiological workup, nearly 50% of the cases remain unexplained. Compared to other diagnostic groups, ALF of unknown aetiology is associated with lower spontaneous survival, higher transplantation and mortality rates in children.^1^, ^2^, ^3^ A recent comprehensive study utilizing WES in total 260 children presented with indeterminate ALF revealed a genetic diagnosis in ~37% of the cases, corresponding to 36 different previously‐known morbid genes.^16^ Of note, six of these 36 genes, *MRPS5*, *SUCLG1*, *AP4M1*, *CACNA1E*, *NSD1* and *STAT3* were not present among our panel of 380 genes curated from the OMIM based on an associated hepatobiliary phenotype, yet we did not find any candidate disease‐causing variation in those six genes in ALF patients in this report. Consistently, previous studies on the genetics of indeterminate paediatric ALF did not reveal an underlying monogenic cause in majority of the cases, whereas some were diagnosed with hitherto unrecognized inherited metabolic diseases.^18^, ^19^, ^20^, ^21^, ^22^, ^23^ It is also possible that nongenetic factors may account for ALF in some children. Nonetheless, WES and/or whole‐genome sequencing (WGS) should be included in the diagnostic workup of idiopathic liver injury, including ALF and persistent/recurrent ELT, in children for earlier identification of the underlying disease aetiology, proper medical management and referral for liver transplantation when necessary. Furthermore, unbiased analyses of WES/WGS data of large numbers of paediatric ALF patients will certainly help in the discovery of novel disease‐causing genetic lesions.

## AUTHOR CONTRIBUTIONS

**Aysima Atılgan Lülecioğlu:** Data curation (supporting); formal analysis (equal); investigation (equal); validation (equal); writing – original draft (equal); writing – review and editing (supporting). **Yılmaz Yücehan Yazıcı:** Data curation (equal); formal analysis (equal); investigation (equal); methodology (supporting); validation (equal). **Alperen Baran:** Formal analysis (supporting); investigation (supporting); validation (supporting). **Khaled Warasnhe:** Resources (supporting); writing – review and editing (supporting). **Şengül Beyaz:** Resources (equal); writing – original draft (supporting); writing – review and editing (supporting). **Caner Aytekin:** Resources (equal); writing – review and editing (supporting). **Figen Özçay:** Resources (equal); writing – review and editing (supporting). **Yusuf Aydemir:** Resources (equal); writing – review and editing (supporting). **Zeren Barış:** Resources (equal); writing – review and editing (supporting). **Serkan Belkaya:** Conceptualization (lead); data curation (equal); formal analysis (equal); funding acquisition (lead); investigation (equal); methodology (equal); project administration (lead); resources (lead); supervision (lead); validation (equal); writing – original draft (equal); writing – review and editing (equal).

## CONFLICT OF INTEREST STATEMENT

The authors declare no conflict of interest.

## Supporting information


Data S1.

## Data Availability

Data related to this study are available upon request from the corresponding author.
