# Supplementary material for: Whole‐exome sequencing for genetic diagnosis of idiopathic liver injury in children
Source: J Cell Mol Med. 2024 Jun 12;28(11):e18485. doi: 10.1111/jcmm.18485 (PMC11167704; doi:10.1111/jcmm.18485)
Supplement: Supplementary file 1 — Data S1. [file JCMM-28-e18485-s001.pdf]

Electronic Supplementary Material

Whole-exome sequencing for genetic diagnosis of idiopathic liver injury in children

Aysima Atılgan Lülecioğlu[1], Yılmaz Yücehan Yazıcı[1], Alperen Baran[1], Khaled Warasnhe[2], Şengül Beyaz[3], Caner Aytekin[4], Figen Özçay[5], Yusuf Aydemir[6], Zeren Barış[6], and Serkan Belkaya[1,*]

[1]Department of Molecular Biology and Genetics, Faculty of Science, İhsan Doğramacı Bilkent University, Ankara, Turkey
[2]Department of Pediatrics, Başkent University Faculty of Medicine, Ankara, Turkey
[3]Department of Immunology and Allergy Diseases, Ankara Bilkent City Hospital, Ankara, Turkey
[4]Department of Pediatric Immunology, Dr. Sami Ulus Maternity and Children's Health and Diseases Training and Research Hospital, Ankara, Turkey
[5]Department of Pediatric Gastroenterology and Hepatology, Başkent University Faculty of Medicine, Ankara, Turkey
[6]Department of Pediatric Gastroenterology, Faculty of Medicine, Eskişehir Osmangazi University, Eskişehir, Turkey

*Correspondence
Serkan Belkaya, PhD
E-mail: sbelkaya@bilkent.edu.tr

This electronic supplementary file includes:

Supplemental Materials and Methods

Supplemental Tables 1 and 2

Supplemental Figure 1

Supplemental References

Supplementary Materials and Methods

WES analysis and variant filtering

Exome capture was performed using SureSelect Human All Exon V6 kit (Agilent Technologies, USA) or the Human Comprehensive Exome panel (Twist Bioscience, USA) by Macrogen, Europe, or Genoks, Turkey, respectively. Paired-end sequencing was performed on a NovaSeq 6000 (Illumina, USA), generating 150-base pair (bp) reads. FASTQ generation was done using BCL2Fastq2 v2.20. BWA-MEM was used for the alignment with the reference human genome (GRCh37). Downstream processing was carried out with Picard Tools, GATK, and SnpEff. All variant calls with depth of coverage (DP) <10, mapping quality <40, and genotype quality <30 were filtered out. Homozygous or hemizygous with allelic depth (AD) to DP ratio <0.9 and heterozygous variants with AD/DP <0.25 were removed. Given the rare occurrence of idiopathic liver injury in children, common variants with AF ≥1% in gnomAD v2.1.1, including variants with AF ≥1% in subpopulations (African, Ashkenazi Jewish, Finnish, non-Finnish European, South Asian, East Asian, Latino and Other), were excluded. Polymorphisms (AF ≥1%) in Turkish Genome Project Data Sharing Portal (TGP) (https://tgd.tuseb.gov.tr/), which includes genome data of more than 500 healthy individuals from Turkey, were further eliminated. Moreover, variants listed as blacklist (previously described pre-calculated list of non-pathogenic variants that are common in exomes of patients but absent from public databases) were filtered out.[1]

Table S1. List of primers used in this study

Primer	Sequence	Purpose
PCK2_G215D_Fwd	GCTCACTGACTCAGCCTATG	Sanger sequencing (G215D)
PCK2_G215D_Rev	GACTGCTGCTCTGGATCTTG	Sanger sequencing (G215D)
VPS33B_L403F_Fwd	CCCTGTGTCCTGGTGATAATTC	Sanger sequencing (L403F)
VPS33B_L403F_Rev	GCATTTAGAGGTGGGGTCG	Sanger sequencing (L403F)
ACOX2_R225Q_Fwd	GCACCAAGTCTGTCCAACTG	Sanger sequencing (R225Q)
ACOX2_R225Q_Rev	GGGAAAAGCAATGCACAGGTC	Sanger sequencing (R225Q)
PYGL_E394X_Fwd	GGAACACTGTAGCCATCTGT	Sanger sequencing (E394X)
PYGL_E394X_Rev	GCTGACTCCTTCCTTTCCC	Sanger sequencing (E394X)
ABCB4_A984T_Fwd	GCATCATCAGGCATCAGAGAAC	Sanger sequencing (A984T)
ABCB4_A984T_Rev	GGGGAGAAAGGGGATGATTAAG	Sanger sequencing (A984T)
PHKA2_R186C_Fwd	TCCTGATGAAGGGAACCAAATC	Sanger sequencing (R186C)
PHKA2_R186C_Rev	ACTTATGTTTCAGTGTGGCTGC	Sanger sequencing (R186C)
SLC27A5_T308M_Fwd	CCTCAGAAGTGGGCTTACCG	Sanger sequencing (T308M)
SLC27A5_T308M_Rev	TTCAGCCTGTGAACCCAACC	Sanger sequencing (T308M)
CDAN1_R649W_Fwd	CCAAGGAAACCAGTCAGCTT	Sanger sequencing (R649W)
CDAN1_R649W_Rev	ATGCTTGGACCTTTTACTTCCC	Sanger sequencing (R649W)
JAG1_N108H_Fwd	TCTCGCAAGGGATAACAGGG	Sanger sequencing (N108H)
JAG1_N108H_Rev	CGACGAGTGTGACACATACTTC	Sanger sequencing (N108H)

Fwd: Forward, Rev: Reverse

Table S2. Liver panel genes

#	Gene	Phenotype MIM	Inheritance
1	ABCA1	205400	AR
2	ABCB11	601847; 605479	AR
3	ABCB4	614972	AD, AR
		602347	AR
		600803	AD, AR
4	ABCC2	237500	AR
5	ABCD3	616278	AR
6	ACAD9	611126	AR
7	ACADM	201450	AR
8	ACADVL	201475	AR
9	ACOX1	264470	AR
10	ACOX2	617308	AR
11	ACSF3	614265	AR
12	ACVRL1	600376	AD
13	ADA	102700	AR
14	ADAMTSL2	231050	AR
15	ADK	614300	AR
16	AGA	208400	AR
17	AGL	232400	AR
18	AGPAT2	608594	AR
19	AKR1D1	235555	AR
20	ALAS2	300752	XL
21	ALDOA	611881	AR
22	ALDOB	229600	AR
23	ALG1	608540	AR
24	ALG13	300884	XL
25	ALG2	607906	AR
26	ALG6	603147	AR
27	ALG8	608104	AR
28	ALG9	608776; 263210	AR
29	ALMS1	203800	AR
30	AMACR	214950	AR
31	ANKS6	615382	AR
32	AP1B1	242150	AR
33	AP1S1	609313	AR
34	APOC2	207750	AR
35	ARCN1	617164	AD
36	ARSB	253200	AR
37	ASAH1	228000	AR
38	ASL	207900	AR
39	ASS1	215700	AR
40	ATIC	608688	AR
41	ATP6AP1	300972	XLR
42	ATP6AP2	301045	XLR
43	ATP7B	277900	AR
44	ATP8B1	147480	AD
		211600	AR
		243300	AR
45	B4GALT1	607091	AR
46	BAAT	619232	AR
47	BBS1	209900	AR
48	BCAP31	300475	XLR
49	BCS1L	603358; 124000	AR
50	BLVRA	614156	AD, AR
51	BMP2	235200	AD, AR
52	BOLA3	614299	AR
53	BSCL2	615924; 269700	AR
54	BTD	253260	AR
55	C2ORF69	619423	AR
56	CARS2	616672	AR
57	CASR	239200	AD, AR
58	CAV1	612526	AR
59	CAVIN1	613327	AR
60	CC2D2A	619111; 612284	AR
61	CCDC115	616828	AR
62	CCDC47	618268	AR
63	CDAN1	224120	AR
64	CEP164	614845	AR
65	CEP19	615703	AR
66	CEP41	614464	AR
67	CFTR	219700	AD, AR
68	CIDEC	615238	AR
69	CIITA	209920	AR
70	CLDN1	607626	AR
71	COG2	617395	AR
72	COG4	613489	AR
73	COG5	613612	AR
74	COG6	614576	AR
75	COG7	608779	AR
76	COQ2	607426	AR
77	COX16	619355	AR
78	COX5A	619064	AR
79	CPE	619326	AR
80	CPOX	618892	AD, AR
81	CPT1A	255120	AR
82	CPT2	600649; 608836	AR
83	CREB3L3	619324	AD
84	CSPP1	615636	AR
85	CTNS	219800	AR
86	CYC1	615453	AR
87	CYP7B1	613812	AR
88	DCDC2	616217; 617394	AR
89	DDOST	614507	AR
90	DGUOK	251880; 617068	AR
91	DHDDS	613861	AR
92	DKC1	305000	XLD, XLR
93	DLD	246900	AR
94	DNASE2	619858	AR
95	DPAGT1	608093	AR
96	DPM1	608799	AR
97	DYNC2H1	613091	AR
98	EARS2	614924	AR
99	EFL1	617941	AR
100	EIF2AK3	226980	AR
101	ENG	187300	AD
102	EPM2A	254780	AR
103	ERCC4	615272; 610965	AR
104	ETFA	231680	AR
105	ETFDH	231680	AR
106	F5	600880	AD, AR
107	FADD	613759	AR
108	FAH	276700	AR
109	FAN1	614817	AR
110	FARS2	614946	AR
111	FARSA	619013	AR
112	FARSB	613658	AR
113	FAS	601859	AD
114	FBP1	229700	AR
115	FBXL4	615471	AR
116	FECH	177000	AR
117	FH	606812	AR
118	FOCAD	619991	AR
119	FUCA1	230000	AR
120	G6PC	232200	AR
121	GAA	232300	AR
122	GALE	230350	AR
123	GALM	618881	AR
124	GALNS	253000	AR
125	GALT	230400	AR
126	GANAB	600666	AD
127	GATA6	600001	AD
128	GATC	618839	AR
129	GBA1	608013; 230800; 230900; 231000; 231005	AR
130	GBE1	232500	AR
131	GCDH	231670	AR
132	GFM1	609060	AR
133	GIMAP5	619463	AR
134	GLB1	230600	AR
135	GLIS3	610199	AR
136	GNE	269921	AD
137	GNMT	606664	AR
138	GNPTAB	252500	AR
139	GNS	252940	AR
140	GPC3	312870	XLR
141	GPD1	614480	AR
142	GPIHBP1	615947	AR
143	GUSB	253220	AR
144	HADH	231530	AR
145	HADHA	609016; 609015	AR
146	HADHB	609015	AR
147	HAMP	613313	AR
148	HFE	235200; 176100	AR
149	HGSNAT	252930	AR
150	HJV	602390	AR
151	HMGCL	246450	AR
152	HMGCR	620375	AR
153	HMGCS2	605911	AR
154	HNF1B	137920	AD
155	HNF4A	616026	AD
156	HSD17B13	620116	AD
157	HSD17B4	261515	AR
158	HSD3B7	607765	AR
159	IARS1	617093	AR
160	IDS	309900	XLR
161	IDUA	607014; 607015	AR
162	IER3IP1	614231	AR
163	IFIH1	615846	AD
164	IFT172	615630	AR
165	IFT56	619534	AR
166	IL18BP	618549	AR
167	IL1RN	612852	AR
168	IL21R	615207	AR
169	INPP5E	213300	AR
170	INSR	246200	AR
171	ITCH	613385	AR
172	JAG1	118450	AD
173	JAK1	618999	AD
174	JAK2	600880	AR
175	KARS1	619147	AR
176	KIF12	619662	AR
177	KIF20A	619433	AR
178	KRT18	215600	AR
179	LARS1	615438	AR
180	LARS2	617021	AR
181	LBR	613471	AD
		215140	AR
182	LIPA	278000	AR
183	LIPE	615980	AR
184	LIPT1	616299	AR
185	LMNA	151660	AD
186	LPL	144250	AD
		238600	AR
187	LRPPRC	220111	AR
188	LYN	620376	AD
189	LYRM4	615595	AR
190	MAN1B1	614202	AR

This panel is manually curated by searching for genes annotated with hepatic and/or biliary phenotype or laboratory finding of elevated liver enzymes/transaminases in the OMIM database (https://www.omim.org/). Only protein coding genes were included in the panel. Genes that were associated with somatic morbid mutations were excluded.

Table S2. Liver panel genes (continued)

#	Gene	Phenotype MIM	Inheritance
191	MAN2B1	248500	AR
192	MARS1	615486	AR
193	MCCC2	210210	AR
194	MED12	301068	XLD
195	MICOS13	618329	AR
196	MICU1	615673	AR
197	MKS1	249000	AR
198	MMAA	251100	AR
199	MMAB	251110	AR
200	MMUT	251000	AR
201	MOGS	606056	AR
202	MPI	602579	AR
203	MPV17	618400; 256810	AR
204	MRM2	618567	AR
205	MRPL3	614582	AR
206	MRPL44	615395	AR
207	MRPS16	610498	AR
208	MRPS23	618952	AR
209	MRPS28	618958	AR
210	MRPS7	617872	AR
211	MTM1	310400	XLR
212	MVK	610377	AR
213	MYO5B	619868; 251850	AR
214	NAF1	620365	AD
215	NAGLU	252920	AR
216	NBAS	616483	AR
217	NCF2	233710	AR
218	NCKAP1L	618982	AR
219	NDUFS4	252010	AR
220	NEK8	615415	AR
221	NEU1	256550	AR
222	NFKB1	616576	AD
223	NFS1	619386	AR
224	NGLY1	615273	AR
225	NHLRC1	254780	AR
226	NHLRC2	618278	AR
227	NOP10	620400	AD
		224230	AR
228	NOS3	189800	AD
229	NOTCH2	610205	AD
230	NPC1	257220	AR
231	NPC2	607625	AR
232	NPHP3	267010; 604387; 208540	AR
233	NR1H4	617049	AR
234	OCLN	251290	AR
235	OFD1	311200	XLD
236	OSTM1	259720	AR
237	OTULIN	617099	AD, AR
238	PARS2	618437	AR

#	Gene	Phenotype MIM	Inheritance
239	PC	266150	AR
240	PCCA	606054	AR
241	PCCB	606054	AR
242	PCK1	261680	AR
243	PCK2	261650	AR
244	PCYT1A	620680	AR
245	PEPD	170100	AR
246	PEX1	214100; 601539	AR
247	PEX10	614870	AR
248	PEX12	266510	AR
249	PEX13	614883	AR
250	PEX14	614887	AR
251	PEX16	614876	AR
252	PEX19	614886	AR
253	PEX2	614866; 614867	AR
254	PEX26	614872	AR
255	PEX3	614882	AR
256	PEX5	214110	AR
257	PEX6	614863	AD, AR
		614862	AR
258	PFKM	232800	AR
259	PGM1	614921	AR
260	PHKA2	306000	XLR
261	PHKB	261750	AR
262	PHKG2	613027	AR
263	PIBF1	617767	AR
264	PIGA	300868	XLR
265	PKHD1	263200	AR
266	PLIN1	613877	AD
267	PMM2	212065	AR
268	PNPLA2	610717	AR
269	POLD1	615381	AD
270	POLG	203700; 613662	AR
271	POLG2	619425	AR
		610131	AD
		618528	AR
272	POMC	609734	AR
273	POT1	620367	AD, AR
274	PPARG	604367	AD
275	PRF1	603553	AR
276	PRKCSH	174050	AD
277	PSAP	611721	AR
278	PSMB4	617591	AR
279	PSMB8	256040	AR
280	PSMB9	617591	AR
281	PSMC1	620071	AR
282	PTF1A	615935	AR
283	PYGL	232700	AR
284	QRSL1	618835	AR

#	Gene	Phenotype MIM	Inheritance
285	RBCK1	615895	AR
286	RFT1	612015	AR
287	RFX5	209920	AR
288	RFX6	615710	AR
289	RFXANK	209920	AR
290	RFXAP	209920	AR
291	RINT1	618641	AR
292	RMND1	614922	AR
293	RNASEH2A	610333	AR
294	RNF220	619688	AR
295	RPGRIP1L	619113; 611561	AR
296	RRAGC	620609	AD
297	RTEL1	616373	AD, AR
298	SBDS	260400	AR
299	SC5D	607330	AR
300	SCO1	619048	AR
301	SCYL1	616719	AR
302	SDHD	619167	AR
303	SEC23B	224100	AR
304	SEC63	617004	AD
305	SEMA7A	619874	AR
306	SERAC1	614739	AR
307	SERPINA1	613490	AR
308	SGSH	252900	AR
309	SH2D1A	308240	XLR
310	SKIC2	614602	AR
311	SKIC3	222470	AR
312	SLC10A1	619256	AR
313	SLC17A5	269920	AR
314	SLC22A5	212140	AR
315	SLC25A1	615182	AR
316	SLC25A13	603471; 605814	AR
317	SLC25A15	238970	AR
318	SLC25A20	212138	AR
319	SLC27A5	619232	AR
320	SLC2A1	608885	AD
321	SLC2A2	227810	AR
322	SLC30A10	613280	AR
323	SLC37A4	619525	AD
		232220	AR
324	SLC39A4	201100	AR
325	SLC39A7	619693	AR
326	SLC40A1	606069	AD
327	SLC44A1	618868	AR
328	SLC51A	619484	AR
329	SLC51B	619481	AR
330	SLC7A7	222700	AR
331	SMPD1	257200; 607616	AR
332	SOCS1	619375	AD

#	Gene	Phenotype MIM	Inheritance
333	SPTB	616649	AD
334	SQOR	619221	AR
335	SRD5A3	612379	AR
336	STAT2	618886	AR
337	STT3B	615597	AR
338	STX5	620454	AR
339	STXBP2	613101	AR
340	SUMF1	272200	AR
341	TALDO1	606003	AR
342	TANGO2	616878	AR
343	TEFM	620451	AR
344	TERT	613989	AD, AR
		614742	AD
345	TFAM	617156	AR
346	TFR2	604250	AR
347	TJP2	615878; 607748	AR
348	TKFC	618805	AR
349	TMEM107	617562	AR
350	TMEM165	614727	AR
351	TMEM199	616829	AR
352	TMEM67	216360; 610688; 607361; 613550	AR
353	TRAPPC11	615356	AR
354	TREX1	192315	AD
		225750	AD, AR
355	TRIM37	253250	AR
356	TRMT10C	616974	AR
357	TRMU	613070	AR
358	TTC26	619534	AR
359	TTC7A	243150	AR
360	TULP3	619902	AR
361	TWNK	271245	AR
362	UBR1	243800	AR
363	UNC13D	608898	AR
364	UNC45A	619377	AR
365	UQCRB	615158	AR
366	UQCRC2	615160	AR
367	UROD	176100	AD, AR
368	USP18	617397	AR
369	USP53	619658	AR
370	VIPAS39	613404	AR
371	VPS33B	208085; 620010; 620009	AR
372	VPS50	619685	AR
373	VPS51	618606	AR
374	WDR19	614378; 614377; 616307; 614376	AR
375	WDR35	613610; 614091	AR
376	XIAP	300635	XLR
377	YARS1	619418	AD, AR
378	YARS2	613561	AR
379	YRDC	619609	AR
380	ZNFX1	619644	AR

Supplemental Figure 1

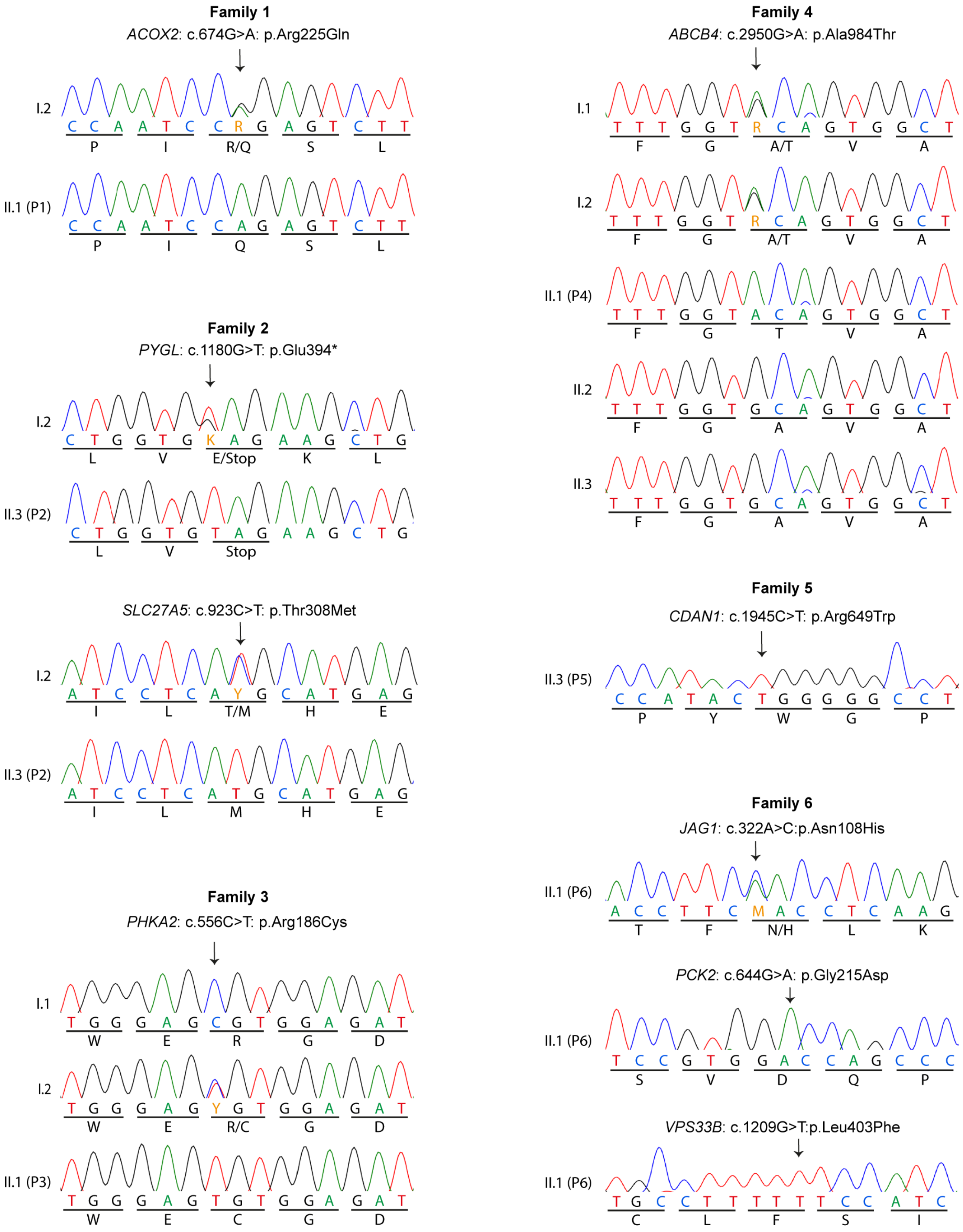

Figure S1. Sanger sequencing of variants in 9 liver panel genes identified in 6 unrelated patients affected with idiopathic liver injury. Carrier testing for some family members was not performed, as their gDNA samples were not available.

Supplemental References

1. Maffucci P, Bigio B, Rapaport F, et al. Blacklisting variants common in private cohorts but not in public databases optimizes human exome analysis. *Proc Natl Acad Sci U S A* 2019; **116**(3): 950-9.
